# Supplementary material for: Dual-Emitting Molecularly Imprinted Nanopolymers for the Detection of CA19-9
Source: Biomedicines. 2025 Jul 3;13(7):1629. doi: 10.3390/biomedicines13071629 (PMC12292350; doi:10.3390/biomedicines13071629)
Supplement: Supplementary file 1 [file biomedicines-13-01629-s001.zip › biomedicines-3649176-supplementary.pdf]

Supplementary Material (SM)

to

Dual-Emitting Molecularly Imprinted Nanopolymers for the Detection of CA19-9

Eduarda Rodrigues [1], Ana Xu [1], Rafael C. Castro [2], David S. M. Ribeiro [2], João L. M. Santos [2] and Ana Margarida L. Piloto [1,2,*]

[1] CIETI-LabRISE, ISEP, Polytechnic of Porto, Rua Dr. António Bernardino de Almeida 431, 4249-015 Porto, Portugal; edmfr@isep.ipp.pt (E.R.); naaxu@isep.ipp.pt (A.X.)

[2] LAQV, REQUIMTE, Laboratory of Applied Chemistry, Department of Chemical Sciences, Faculty of
Pharmacy, University of Porto, Rua de Jorge Viterbo Ferreira nº 228, 4050-313 Porto, Portugal; rafael.castro.cl@hotmail.com (R.C.C.); dsmribeiro@gmail.com (D.S.M.R.); joaolms@ff.up.pt (J.L.M.S.)

* Correspondence: amlpc@isep.ipp.pt

Table of contents

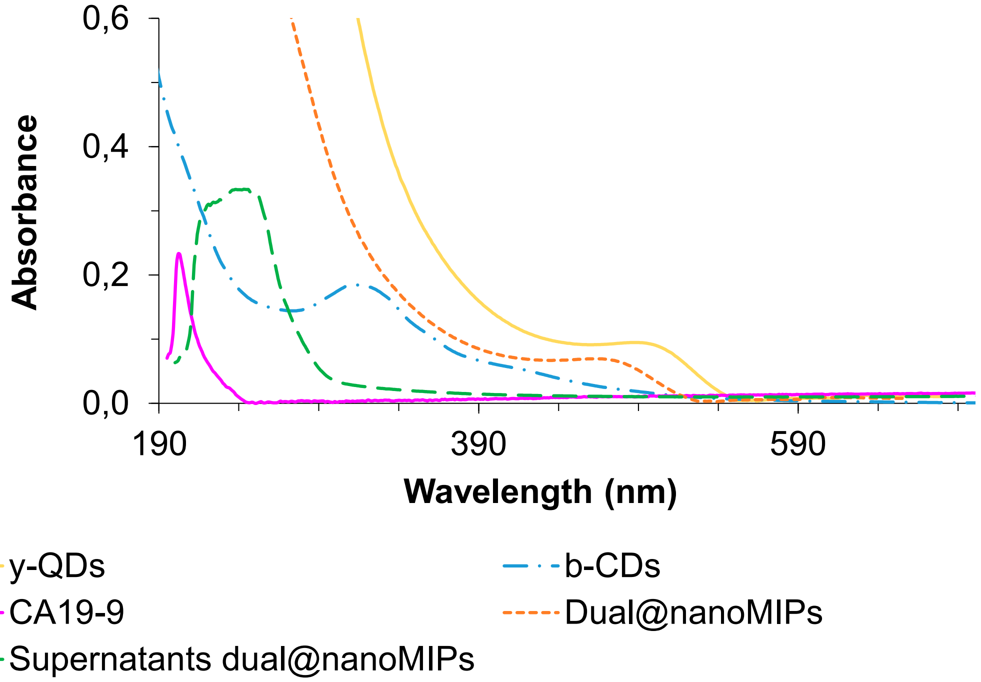

Figure S1. UV/vis spectra during assembly of the dual@nanoMIPs. y-QDs at 1 mg mL⁻¹ (yellow solid line); b-CDs stock solution diluted in PBS (Abs(λ_{max} 320) = 0.2) (blue dot and dash line); dual@nanoMIPs after washings (orange dash line); CA19-9 antigen at 10 kU mL⁻¹ (pink solid line); supernatants of dual@nanoMIPs (green dash line). All spectra were recorded in 10 mM PBS at pH 7.4.

Quantum Yield (QY) calculations

The quantum yields (QYs) of the synthesized b-CDs and y-QDs were determined using a comparative method with well-established reference fluorophores. For b-CDs, quinine sulfate in 0.1 M H_2SO_4 ($\Phi_r = 0.54$) was used as the reference standard, while for y-QDs, rhodamine 6G in ethanol ($\Phi_r = 0.95$) was employed. All solutions were prepared to have absorbance values below 0.1 at the excitation wavelength (**λ_{ex} = 320 nm**) for b-CDs and (**λ_{ex} = 390 nm**) for y-QDs to minimize inner filter effects. All spectra were recorded at room temperature (22 °C). The absorbance of both sample and standard was kept below 0.1 at their corresponding excitation wavelengths to minimize inner filter effects. Solvent: Milli-Q water.

Fluorescence emission spectra were recorded under identical conditions, and the integrated emission intensities were used in the following equation:

$$\Phi_x = \Phi_r \left(\frac{I_x}{I_r}\right)\left(\frac{A_r}{A_x}\right)\left(\frac{n_x^2}{n_r^2}\right)$$

Where:

Φ_x is the quantum yield of the sample (b-CDs or y-QDs);

Φ_r is the quantum yield of the reference fluorophore (standard):

For b-CDs: Quinine Sulfate in 0.1 M H_2SO_4 (Φ = 0.54)
For y-QDs: Rhodamine 6G in ethanol (Φ = 0.95)

I_x and I_r are integrated fluorescence intensities of the sample and reference

A_x and A_r are absorbances at the excitation wavelength (kept below 0.1 to avoid inner filter effects);

n_x and n_r are the refractive indices of solvents for sample and reference (usually the same if both in water or PBS), (both water, so $\frac{n_x^2}{n_r^2} = 1$).

Experimental Conditions

All spectra were recorded at room temperature (22 °C)
Absorbance values were kept below 0.05 to minimize inner filter effects.
Solvents used: 0.1 M H_2SO_4 for quinine sulfate (n ≈ 1.33), ethanol for Rhodamine 6G (n ≈ 1.36), and PBS (n ≈ 1.33) for sample measurements.
All spectra were corrected for baseline and instrument response.
QY values and its standard deviation were averaged in triplicate, as shown in **Table S1**.

Results

The QY of b-CDs was calculated as 22.6 ± 1.4% using quinine sulfate as the reference.
The QY of y-QDs was calculated as 31.2 ± 2.3% using rhodamine 6G as the reference.

Table S1. Relative Quantum Yield Determination of b-CDs and y-QDs.

Sample	Reference Fluorophore	Φ_r	λ_{ex} (nm)	A_{sample}	A_{ref}	I_{sample}	I_{ref}	Solvent (n)	Φ (%)	Std. Dev. (n = 5)
b-CDs	Quinine sulfate	0.54	320	0.045	0.046	382.4	910.1	1.33	22.6	±1.4
y-QDs	Rhodamine 6G	0.95	390	0.042	0.044	679.2	2069.5	1.36	31.2	±2.3

Stern-Volmer plots for calibrations of the dual@nanoMIPs in PBS

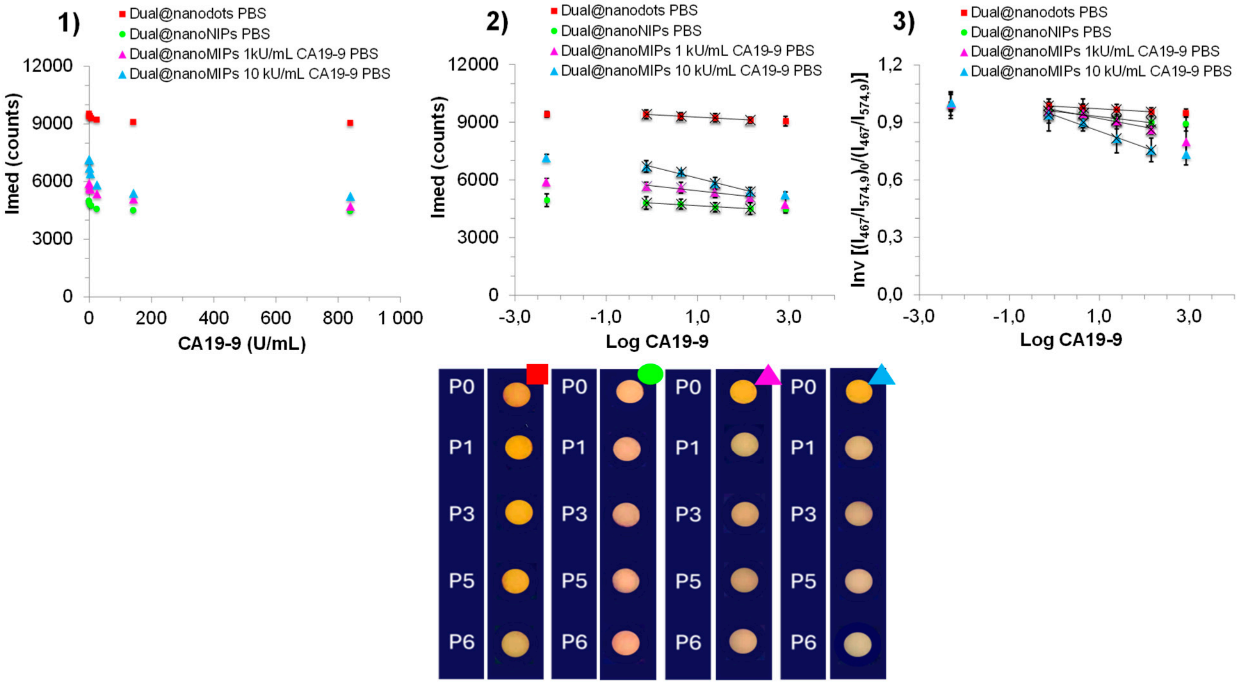

Figure S2. Stern-Volmer plots for calibrations of the dual@nanoMIPs in PBS: dual@nanodots (red), dual@nanoNIPs (green) and with dual@nanoMIPs imprinted with 1 kU mL^{-1} CA19-9 (pink dots) and with 10 kU mL^{-1} CA19-9 (blue dots). Calibrations were performed with CA19-9 standards ranging from 4.98×10^{-3} U mL^{-1} to 8.39×10^{2} U mL^{-1} in PBS. The analytical data is shown in **Table S2**.

Table S2. Analytical data of the dual@nanodots, of the dual@nanoMIPs and of the dual@nanoNIPs, upon calibrations with standards of CA19-9 within the range 4.98×10^{-3} U mL^{-1} to 8.39×10^{2} U mL^{-1} in PBS. Limit of detection (LOD), Stern-Volmer constant (k_{SV}) and imprinting factor (IF).

Data	dual@ nanodots	dual@ nanoMIPs 1 kU/mL	dual@ nanoMIPs 10 kU/mL	dual@ nanoNIPs
LOD (U/mL)	4.36×10^{-3}	$1.59\times10^{-3-}$	1.20×10^{-3}	1.21×10^{-2}
k_{SV}	-0.0135	-0.0443	-0.0837	-0.0275
IF	…	1.61	3.04	…

Stern-Volmer plots for calibrations of the dual@nanoMIPs in 1% HN serum in PBS

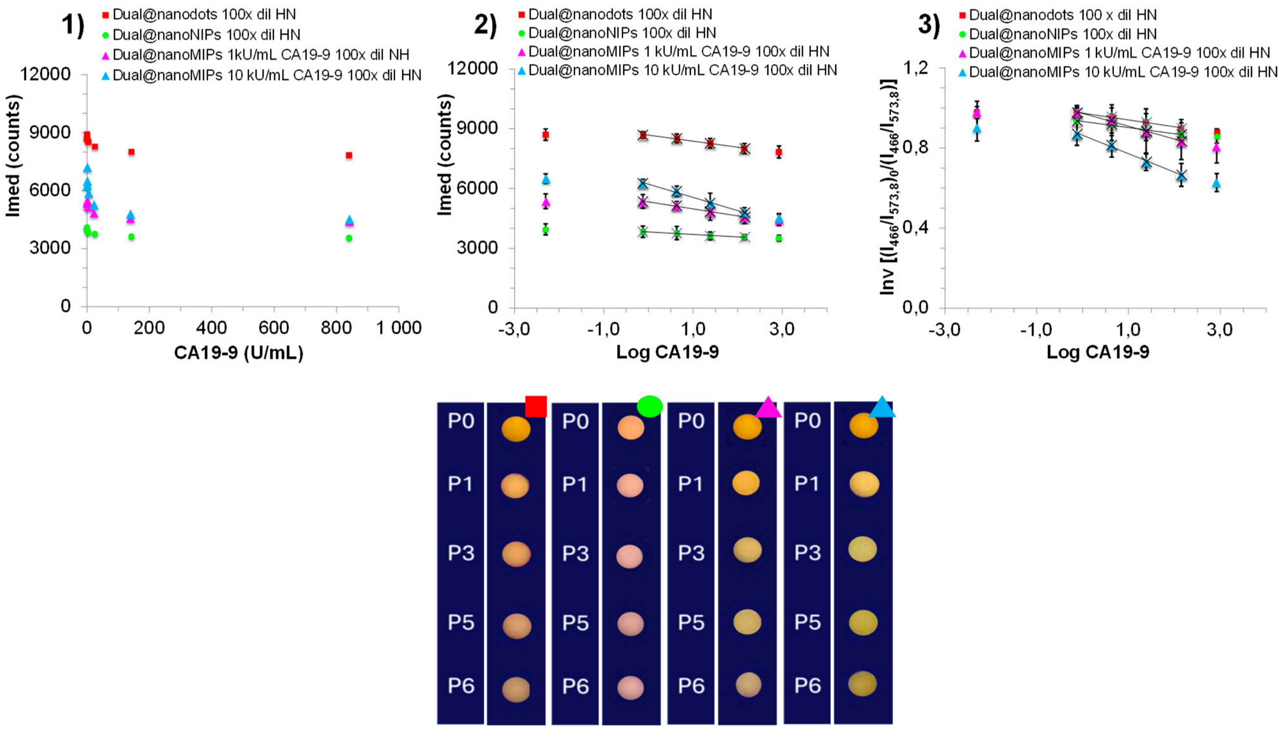

Figure S3. Stern-Volmer plots for calibrations of the dual@nanoMIPs in 1% HN serum in PBS: dual@nanodots (red), dual@nanoNIPs (green), and dual@nanoMIPs imprinted with 1 kU/mL CA19-9 (pink dots) and with 10 kU/mL CA19-9 (blue dots). Calibrations were performed with CA19-9 standards ranging from 4.98×10^{-3} U mL^{-1} to 8.39×10^{2} U mL^{-1} in 1% HN serum in PBS. The analytical data is shown in **Table S3**.

Table S3. Analytical data of the dual@nanodots, of the dual@nanoMIPs and of the dual@nanoNIPs, upon calibrations with standards of CA19-9 within the range 4.98×10^{-3} U mL^{-1} to 8.39×10^{2} U/ mL^{-1} in 1% HN serum in PBS 10 mM pH 7.4. Limit of detection (LOD), Stern-Volmer constant (k_{SV}) and imprinting factor (IF).

Data	dual@ nanodots	dual@ nanoMIPs 1 kU/mL	dual@ nanoMIPs 10 kU/mL	dual@ nanoNIPs
LOD (U/mL)	3.97×10^{-3}	$3.33 \times 10^{-3-}$	2.40×10^{-3}	4.83×10^{-3}
k_{SV}	0.0340	-0.0643	-0.0942	-0.0305
IF	…	2.10	3.01	…
